# Long-term effects of asthma medication on asthma symptoms: an application of the targeted maximum likelihood estimation

**DOI:** 10.1186/s12874-020-01175-9

**Published:** 2020-12-16

**Authors:** Carolin Veit, Ronald Herrera, Gudrun Weinmayr, Jon Genuneit, Doris Windstetter, Christian Vogelberg, Erika von Mutius, Dennis Nowak, Katja Radon, Jessica Gerlich, Tobias Weinmann

**Affiliations:** 1 Institute and Clinic for Occupational, Social and Environmental Medicine, LMU University Hospital Munich, Munich, Germany; 2 Department of Medical Informatics, Biometry and Epidemiology (IBE), Ludwig-Maximilian University Munich (LMU), Munich, Germany; 3 Comprehensive Pneumology Center CPC LMU Munich, member of the German Center for Lung Research (DZL), Munich, Germany; 4 Institute of Epidemiology and Medical Biometry, Ulm University, Ulm, Germany; 5 Paediatric Epidemiology, Hospital for Children and Adolescents, University of Leipzig Medical Center, Leipzig, Germany; 6 Paediatric Department, University Hospital Carl Gustav Carus Dresden, TU Dresden, Dresden, Germany; 7 Dr. v. Haunersches Kinderspital, LMU University Hospital Munich, Munich, Germany

**Keywords:** Adolescents, Asthma, Children, Control medication, Marginal structural models, Targeted-maximum likelihood estimation

## Abstract

**Background:**

Long-term effectiveness of asthma control medication has been shown in clinical trials but results from observational studies with children and adolescents are lacking. Marginal structural models estimated using targeted maximum likelihood methods are a novel statistiscal approach for such studies as it allows to account for time-varying confounders and time-varying treatment. Therefore, we aimed to calculate the long-term risk of reporting asthma symptoms in relation to control medication use in a real-life setting from childhood to adulthood applying targeted maximum likelihood estimation.

**Methods:**

In the prospective cohort study SOLAR (Study on Occupational Allergy Risks) we followed a German subsample of 121 asthmatic children (9–11 years old) of the ISAAC II cohort (International Study of Asthma and Allergies in Childhood) until the age of 19 to 24. We obtained self-reported questionnaire data on asthma control medication use at baseline (1995–1996) and first follow-up (2002–2003) as well as self-reported asthma symptoms at baseline, first and second follow-up (2007–2009). Three hypothetical treatment scenarios were defined: early sustained intervention, early unsustained intervention and no treatment at all. We performed longitudinal targeted maximum likelihood estimation combined with Super Learner algorithm to estimate the relative risk (RR) to report asthma symptoms at SOLAR I and SOLAR II in relation to the different hypothetical scenarios.

**Results:**

A hypothetical intervention of early sustained treatment was associated with a statistically significant risk increment of asthma symptoms at second follow-up when compared to no treatment at all (RR: 1.51, 95% CI: 1.19–1.83) or early unsustained intervention (RR:1.38, 95% CI: 1.11–1.65).

**Conclusions:**

While we could confirm the tagerted maximum likelihood estimation to be a usable and robust statistical tool, we did not observe a beneficial effect of asthma control medication on asthma symptoms. Because of potential due to the small sample size, lack of data on disease severity and reverse causation our results should, however, be interpreted with caution.

## Background

Asthma is a chronic inflammatory disease, which affects the respiratory system and leads to different symptoms varying from one individual to another and across time. These include wheezing, shortness of breath, cough, chest tightness and impaired lung function because of constriction and inflammation of the bronchial system [1]. The disease is ranked as the 16th most leading cause of years lived with disability (YLD) worldwide [2]. Its prevalence varies depending on country [3] as well as on the definition of asthma [4]. Furthermore, asthma is the most common chronic disease among children [5]. According to the ISAAC study, the prevalence of this disease ranges between six and 27% in adolescents aged between 13 and 14 years [3]. As asthma often develops during childhood, early intervention should aim to prevent a decrease or impaired increase in lung function until adulthood [6].

The pathogenesis of different phenotypes of asthma is still not fully elucidated [7] with guideline interventions in the form of pharmacological treatment mainly targeting the control of symptoms rather than curing the initial cause of the disease [1]. Asthma treatment recommended by the Global Initiative for Asthma (GINA) is divided into reliever medication, which is indicated for acute asthma attacks, and control medication, which aims to control symptoms and prevent asthma exacerbations [1].

Since asthma is a chronic disease, long-term outcomes are of particular interest to evaluate the effectiveness of interventions. Several clinical trials have studied the long-term effect of different asthma medications on symptom control and lung function, finding a positive effect especially for inhaled corticosteroids (ICS) [8–12]. Nevertheless, clinical randomised controlled trials (cRCTs) may not always represent the general population because the study population is highly selected based on various criteria [13]. Consequently, evidence for treatment guidelines should be a combination of results from cRCTs and real-life studies, such as observational studies [14]. However, there are only few observational studies investigating this study question. In some of them, the authors concluded that asthma control did not reach the desired level that better asthma management would increase asthma control [15, 16]. This emphasises that a positive effect of asthma medication in a cRCT does not necessarily result in a positive effect in real-life circumstances.

To analyse observational data, traditional regression methods are often used. However, standard regression analyses like logistic regression are not suitable in case of time-varying treatment and confounding, which can occur in longitudinal studies [17]. An alternative approach to study longitudinal data in presence of time-varying confounding is provided by structural models [17] estimated using targeted maximum likelihood estimation (TMLE) [18, 19]. This approach has already been applied to different longitudinal settings and its desirable statistical properties have been shown [20–22]. It allows interpreting the effect of hypothetical intervention scenarios as it is done in randomised controlled trials adjusted for time-varying confounders. The main difference compared to traditional regression analyses is that no assumption about the distribution of the data structure is made [19]. If these assumptions are violated, which is the case when time-varying confounding occurs, traditional regression methods will lead to biased results [19].

To our knowledge, no previous study has investigated the long-term effect of asthma control medication on symptoms using the TMLE approach. Furthermore, only few studies included children and adolescents [23]. Therefore, we aimed to apply this strategy to study the long-term effect of hypothetical asthma treatment regimens on symptoms of children and adolescents. Based on the results of cRCTs, our hypothesis was that early asthma medication should improve symptoms in children and young adults.

## Methods

### Study design and study population

**Fig. 1:**
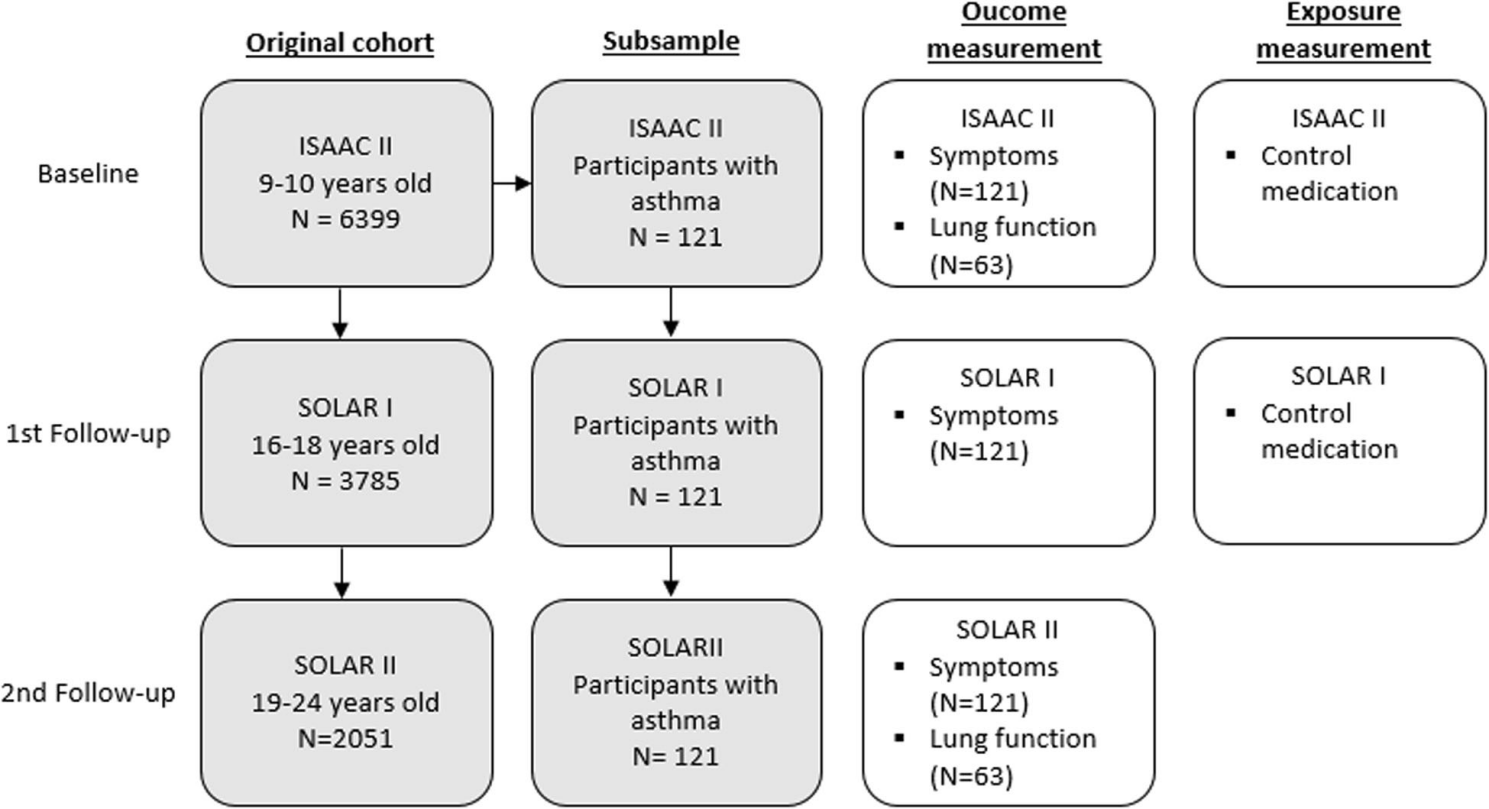
Flow chart for the original cohort and the subsample of participants with asthma from baseline (ISAAC II) to first follow-up (SOLAR I) and second follow-up (SOLAR II) (grey boxes). Time points for outcome and exposure measurements are shown in the white boxes

Our cohort consists of a subsample of asthmatic participants of the ISAAC II study in Munich and Dresden, which took place in 1995–1996 and had two follow-ups by SOLAR I in 2002–2003 and by SOLAR II in 2007–2009. Manuscripts describing the respective study designs are available elsewhere [24–26]. In short, 6399 parents of children aged nine to eleven that were recruited via random sampling filled in a standardised questionnaire for their offspring in ISAAC II (response: 85%) and 2589 of those children took part in a clinical assessment including spirometry. By the age of 16 to 18 years, 3785 participants of the original ISAAC II cohort filled in the follow-up questionnaire (response: 80%). At the second follow-up, 2051 participants aged 19 to 24 years completed the questionnaire (response: 71%) with 57% of them taking part in clinical examinations including spirometry (SOLAR II) (Fig. 1). Out of those 2051 participants, we selected those individuals that were classified as asthmatics at baseline (ISAAC II) as our study population for the present analysis (*n* = 121, Fig. 1). This selection is due to the fact that the main interest of this analysis was the effect of asthma medication on asthma symptoms.

The three questionnaires were based on validated items from ISAAC [27] and the European Community Respiratory Health Survey (ECRHS) [28]. They contained questions on sociodemographic data, respiratory symptoms, allergic symptoms, as well as environmental and occupational risk factors. Additionally, at baseline and first follow-up, the participants provided self-reported data on medication use in case of respiratory problems. Clinical assessment at ISAAC II and SOLAR II included, among others, spirometry and bronchial hyperresponsiveness testing (BHR) with saline.

### Outcome of interest

Our outcome of interest was the presence of asthmatic symptoms in the previous twelve months. Following the GINA Guideline [1], we defined the following items to measure asthma symptoms: daytime symptoms (cough or wheezing), nighttime symptoms (awaking due to wheezing or breathlessness) and limitation of activity (breathlessness at rest or after physical activity). Data on symptoms were available for all three time points (Fig. 1) and a binary variable was created as either having reported symptoms or not. Reliever medication intake is another criterion of asthma control in the GINA guideline. For SOLAR II it was, however, not measured. Therefore, we only used the symptom criteria of the GINA Guidline.

For sensitivity analysis, we defined lung function as a continuous variable. We calculated the ratio of *forced expiratory volume in one second* to *forced vital capacity (FEV*_*1s*_ */ FVC)*, as it is described as an indicator for obstructive airway diseases in the Report of the Global Lung Initiative [29].

### Exposure definition

We defined a binary exposure variable for self-reported control medication intake in the last twelve months (yes versus no). The exposed group contained those who reported the intake of control medication whereas the unexposed group included those who reported no medication use or only reliever medication use. Self-reported medication was assigned to active agents and drug classes based on the Drug Information System from the German Institute of Medical Documentation [30], the Anatomical Therapeutic Chemical (ATC) classification system [31] and the GINA guideline [1] (Table 1).

**Table 1:** Classification of reliever medication and control medication based on active agents and drug classes

	Drug class	Active agent
**Reliever medication**	Short-acting β2-agonists (SABA)	Terbutaline, salbuatmol, tulobuterol, fenoterol, epinephrine/ adrenaline
	Combinations of SABA and mast-cell stabilizers	Reproterol + sodiumcromoglicat, fenoterol + cromoglicic acid
	Short-acting muscarinic antagonists (SAMA)	Ipratropium bromide
	Combinations of SABA and SAMA	Fenoterol + ipratropium bromide
**Control medication**	Inhaled corticosteroids (ICS)	Beclomethasone, fluticasone, budesonide, dexamethasone, flunisolide
	Long-acting β2-agonists (LABA)	Formoterol, salmeterol, clenbuterol
	Combinations of ICS and LABA	Fluticasone + salmeterol, formoterol + budesonide
	Leukotriene receptor antagonists	Montelukast
	Systemic corticosteroids	Betamethasone, cortisone, prednisone, prednisolone, mometasone
	Mast-cell-stabilizer	Cromocligic acid
	Methylxanthines	Methylxanthines, aminophylline

### Hypothetical intervention scenarios

We defined different types of hypothetical intervention scenarios (Table 2). First, we were interested in a hypothetical scenario in which none of the participants had taken control medication neither at ISAAC II nor at SOLAR I (Intervention 1: “0,0”). Hypothetical Intervention 2 consisted of patients taking control medication only at ISAAC II (“1,0”). Intervention 3 describes a scenario in which everyone had taken control medication at both time points (“1,1”). No intervention means that the pattern of control medication intake is as it occurs in the data. We chose these types of interventions because we could then later compare the effects of sustained treatment (Intervention 3), early but not sustained treatment (Imtervention 2), and no treatment at all (Intervention 1). We did not include a scenario for late intervention because we were interested in the long-term effect of early intervention.

**Table 2:** Description of defined hypothetical intervention scenarios

Type of Intervention	Description
Intervention 1 (0,0)	No one takes control medication neither at ISAAC II nor at SOLAR I
Intervention 2 (1,0)	Everyone takes control medication at ISAAC II but not at SOLAR I
Intervention 3 (1,1)	Everyone takes control medication both at ISAAC II and SOLAR I
No Intervention	Control medication is taken as it was observed in the data

### Potential confounders

**Fig. 2:**
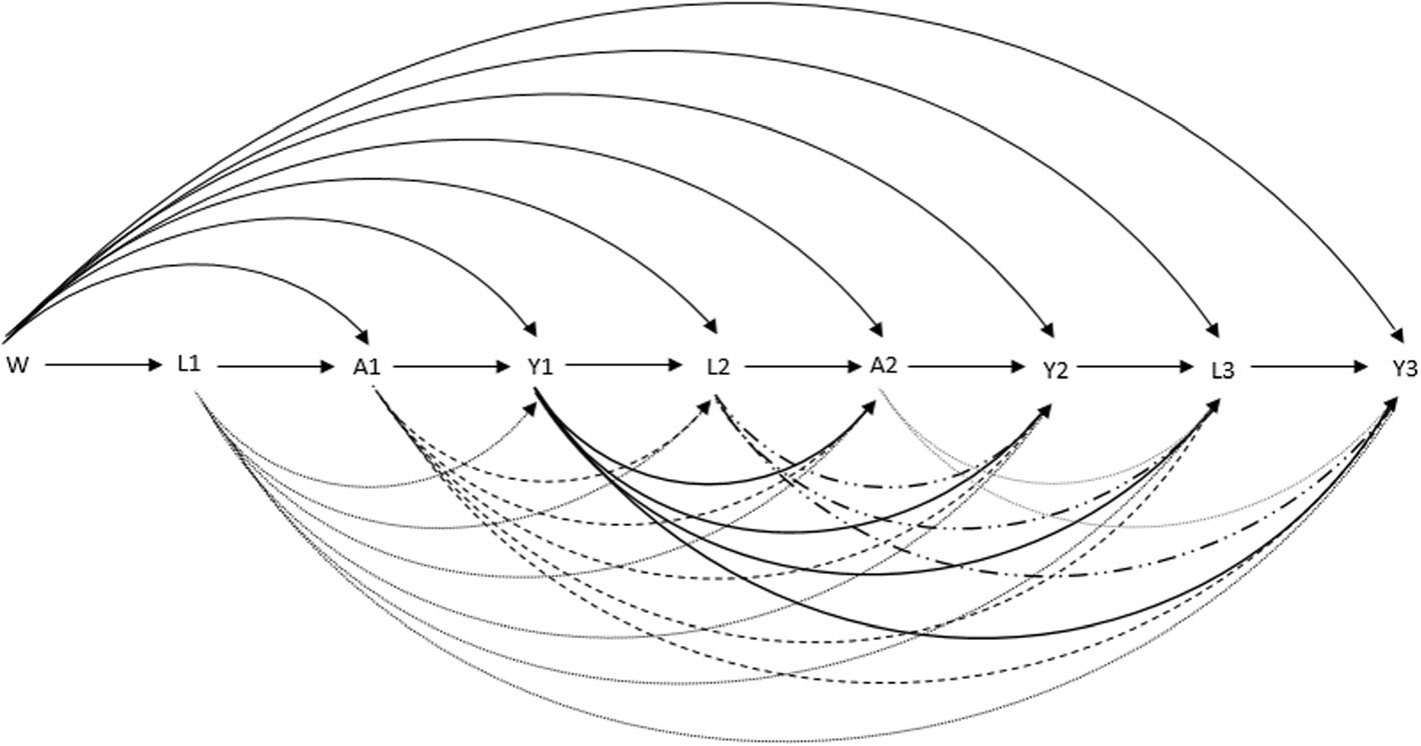
Potential causal relationship between the included covariates. **Legend:** W = Baseline covariates (ses, sex, study centre, parental asthma history); L1, L2, L3 = time varying confounders at ISAAC II, SOLAR I, SOLAR II (hay fever, overweight, smoking, passive smoking, age); A1, A2 = Treatment at ISAAC II and SOLAR I; Y1, Y2, Y3 = intermediate outcomes and final outcome (asthma symptoms) at ISAAC II, SOLAR I, SOLAR II

The selection of the confounders was based on prior knowledge from earlier studies and their potential relation ship was depicted in a causal diagram (Fig. 2). To this end, we differentiated between baseline confounders (W) and time-varying confounders (L1, L2, L3) at ISAAC II, SOLAR I, SOLAR II. In particular, as potential baseline confounders we considered sex (male vs. female), parental socio-economic status (high, vs. low), study center (Munich or Dresden) and parental asthma history (yes vs. no) which might be related to asthma treatment (A) and the outcome (Y). As time-varying binary confounders (L), we included current hay fever (yes vs. no; ever diagnosed hay fever by a doctor and itchy eyes and runny nose without cold in the last 12 months), physical activity (yes vs. no; participant reports excercising at least once per week), smoking status (smoker vs. non-smoker), second hand smoke (yes vs. no) and overweight (body mass index (bmi) > = 25 kg/m^2^ vs. bmi < 25 kg/m^2^). Furthermore, we took age into account as a continuous covariate [32–35]. Additionally, for the sensitivity analysis regarding the outcome, we considered height as continuous covariate because it is a predictor of lung function.

### Statistical analysis

Marginal structural models (MSM) are a suitable statistical approach for longitudinal data in which time-varying treatment and confounding occurs [17]. Therefore, we used TMLE combined with Super Learner algorithm [36] to estimate the relative risk (RR) to report asthma symptoms at SOLAR I and SOLAR II in relation to the different hypothetical scenarios (Table 2).

To identify causal effects via MSM it is necessary to state certain assumptions. Firstly, this comprises the consistency assumption [37], which means that the observed outcome (asthma symptoms yes vs. no) of an individual, taking control medication or not is the same as the counterfactual outcome under control medication or no control medication. Secondly, we assume conditional exchangeability [38], indicating that we included all reasonably possible confounders. The third assumption is positivity [39], stating that there is a probability of greater than zero to be exposed given all combinations of covariates.

For the analyses, we used R version 3.4.3 [40], the package ltmle version 1.0–1 [41] and SuperLearner version 2.0–23 [42]. Furthermore, we assumed that missing values were missing at random. Therefore, we performed multiple imputation (m = 5 imputations) by using the R package mice version 2.46.0 [43] and applying Rubin’s rule [44] to get combined estimators for the relative risks including confidence intervals from the imputed data.

### Sensitivity analyses

Spirometry data was available for ISAAC II and SOLAR II (Fig. 1; Additional file 1). Furthermore, the study population for clinical examination comprised only 63 participants because not all of the participants agreed to take part in clinical measurements. However, we performed a sensitivity analysis with lung function (FEV_1s_ / FVC) as outcome to make a comparison of the estimation approach using self-reported symptoms as outcome with an objective measurement. In contrast to the main analysis in which we calculated relative risks, we calculated the additive treatment effect for the outcome lung function. This was necessary because of the FEV_1s_ / FVC ratio being a continuous variable instead of a binary one.

In addition, we performed a sensitivity analysis in which we classified reliever medication and control medication as exposure variales and considered non-exposed participants as those not taking any medication. This additional classification was done in order to examine if the effect of control medication changes when adding reliever medication to the exposed group.

## Results

The total study sample consisted of 121 participants. There were more male participants in the study population (66%) than females and about half of the children’s parents had a high socio-economic status. A fifth of the participants’ parents have had a history of asthma. Moreover, about a quarter of the study population had current hay fever at baseline, the prevalence of which increased over the two follow-ups. In ISAAC II, 116 participants reported having had asthma symptoms during the last twelve months. This number decreased to 80 and 79 in the two follow-ups. Regarding control medication intake, the number of participants reporting such intake fell from more than 50% at ISAAC II to only 15% at SOLAR II. Consequently, we observed a rise in the percentage of individuals indicating no medication intake at at all from 19% at ISAAC II to 57% at SOLAR I. With respect to potential confounders, we observed that the number of individuals exposed to passive smoke increased from about a quarter of the participants at baseline to almost 50 % at SOLAR I and II. It is also worthwhile to mention that between the first and the second follow-up more participants became overweight (9% vs. 23%) (Table 3).

**Table 3:** Characteristics of the study participants at the three time points ISAAC II, SOLAR I and SOLAR II (*N* = 121)

Variables	ISAAC II	SOLAR I	SOLAR II
Age, mean (sd^a^)	9.6 (0.56)	17.0 (0.59)	22.3 (0.69)
NAs	0	0	0
Asthma symptoms, N (%)			
Yes	116 (95.9)	80 (66.1)	79 (65.3)
NAs^b^	0	2 (1.7)	1 (0.8)
Control medication, N (%)			
Yes	67 (55.4)	18 (14.9)	n.m.^c^
NAs	11 (9.1)	4 (3.3)	
Only reliever medication			
Yes	19 (15.7)	30 (24.8)	n.m.
NAs	11 (9.1)	4 (3.3)	
No asthma medication intake at all			
Yes	24 (19.8)	69 (57.0)	n.m.
NAs	11 (9.1)	4 (3.3)	
Study center, N (%)			
Munich	68 (56.2)		
NAs	0		
Sex			
Male	80 (66.1)		
NAs	0		
SES^d^ parents, N (%)			
High	63 (52.1)		
NAs	1 (0.8)		
Asthma history of parents^e^, N (%)			
Yes	25 (20.7)		
NAs	13 (10.7)		
Passive smoking^f^, N (%)			
Yes	32 (26.4)	58 (47.9)	55 (45.5)
NAs	3 (2.5)	1 (0.8)	1 (0.8)
Current smoker^g^, N (%)			
Yes	n.m.	38 (31.4)	46 (38.0)
NAs		0	0
Physical activity^h^, N (%)			
Yes	n.m.	96 (79.3)	72 (59.5)
NAs		1 (0.8)	0
Overweight^i^			
yes	n.m.	11 (9.1)	28 (23.1)
NAs		2 (1.7)	0
Current hay fever^j^, N (%)			
Yes	32 (26.4)	37 (30.6)	33 (27.3)
NAs	0	1 (0.8)	1 (0.8)

**Abbreviations and comments.** ^a^Sd standard deviation, ^b^NAs missing values, ^c^n.m. not measured, ^d^SES socio-economic status (high if one parent has at least higher secondary education or university degree); ^e^Yes if at least one parent has ever had asthma; ^f^ISAAC II: passive smoking of child if parents are current smokers, SOLAR I/SOLAR II: Yes if exposition to passive smoke per day > 0,5 h; ^g^Current smoker = smoked ever for 1 year and smoked in the last month; ^h^Yes if participant reports doing sport at least once per week; ^i^Yes if body mass index > = 25; ^j^Yes if ever diagnosed hay fever by a doctor and itchy eyes and runny nose without cold in the last 12 months

About one third of the population reported no control medication intake neither in ISAAC II nor in SOLAR I. About 40% reported to take control medication only in ISAAC II but not in SOLAR I, and only 15% took control medication both in ISAAC II and SOLAR I (Table 4). No data on specific medication use was available for SOLAR II.

**Table 4:** Number and percentage of participants with asthma following the treatment interventions of control medication

Intervention 1 (0,0)	Intervention 2 (1,0)	Intervention 3 (1,1)
42 (34.7)	48 (39.7)	18 (14.9)

**Comments**: Intervention 1 (0,0) no control medication intake neither at ISAAC II nor at SOLAR I; Intervention 2 (1,0) control medication only at ISAAC II not at SOLAR I; Intervention 3 (1,1) control medication intake at ISAAC II and SOLAR I

We observed a statistically significant risk increment, adjusted for all in the methods section mentioned confounders, between control medication use and asthma symptoms at SOLAR I when the two extreme intervention scenarios (Intervention 1 and 3) were compared. In other words, there would have been an increased relative risk of 1.37 (95% CI: 1.02–1.73) to report asthma symptoms at SOLAR I had all participants taken control medication at both time points vs. had no one taken control medication at both time points. This association was stronger when we calculated the outcome until SOLAR II (adjusted RR: 1.51, 95% CI: 1.19–1.83). Additionally, the effect was similar (adjusted RR: 1.38, 95% CI: 1.11–1.65) when we compared the sustained intervention (first and second time point) to the early Intervention (only first time point) and calculated until SOLAR II, but not until SOLAR I. For the other comparisons, there were no statistically significant associations (Table 5).

**Table 5:** Associations between control medication use and asthma symptoms at SOLAR I and SOLAR II obtained through TMLE

Treatment scenario (control medication use)	Adjusted relative risk to report asthma symptoms at SOLAR I (95% CI)	Adjusted relative risk to report asthma symptoms at SOLAR II (95% CI)
Intervention 3 vs. Intervention 1 (1,1) (0,0)	1.37 (1.02; 1.73)	1.51 (1.19; 1.83)
Intervention 3 vs. No Intervention (1,1)	1.21 (0.94; 1.49)	1.28 (1.00; 1.57)
Intervention 3 vs. Intervention 2 (1,1) (1,0)	1.24 (0.96; 1.52)	1.38 (1.11; 1.65)
Intervention 2 vs. Intervention 1 (1,0) (0,0)	1.09 (0.79; 1.40)	1.08 (0.76; 1.41)
Intervention 2 vs. No Intervention (1,0)	0.98 (0.73; 1.23)	0.92 (0.64; 1.21)

Adding reliever medication to the control medication as exposure variable, there was a statistically significant risk increment when we compared sustained intervention with early intervention (SOLAR I: adjusted RR = 1.61, 95% CI: 1.32–1.90; SOLAR II: 1.32, 95% CI: 1.03–1.62) (Additional file 2).

Using spirometric results as outcome, we calculated the adjusted additive treatment effect (ATE) on FEV_1s_ / FVC ratio as mean difference between ISAAC II and SOLAR II. We observed a statistically significant reduction of the adjusted FEV_1s_ / FVC ratio of − 4.48% (CI = − 6.97%; − 1.99%) when we compared sustained intervention to early intervention. This indicates that if everyone had taken control medication at both time points compared to if everyone had taken control medication only at ISAAC II, the lung function (measured as FEV_1s_ / FVC ratio) would have decreased by 4.48%. A similar, but weaker association was found when we calculated lung function for the exposure of the sensitivity analysis (adjusted ATE = − 2.82%, CI -4.93%; − 0.71%) (Additional files 3 and 4).

## Discussion

In the present analysis, we applied the targeted maximum likelihood estimation approach to calculate the relative risk to report asthma symptoms in relation to asthma control medication use in a population-based longitudinal setting with twelve years of follow-up. By using a Marginal Structural Models approach, we could account for time-varying treatment and confounding. We observed an increased relative risk of asthma symptoms following a hypothetical sustained intervention of control medication when compared tono treatment at all or early unsustained intervention.

There are several possible explanations why in our study population the intake of asthma control medication tended to be associated with still reporting symptoms on a long-term follow-up. Firstly, it could be that pharmacological treatment used for symptom control cannot alter the underlying disease process [1, 45]. This was, for example, shown in an analysis of German routine data where immunotherapy, which targets the immunological cause of allergic asthma, was more beneficial regarding the course of asthma than asthma medications [46]. Additionally, we followed participants for twelve years. Hence, our results represent long-term effects, which are not necessarily comparable to positive effects of clinical trials with shorter follow-up periods [8–12]. Hence, it may be especially interesting if in the future clinical trials could follow-up their participants for longer periods as to ensure a better comparability with long-standing observational studies such as ours (being fully aware though that such clinical trials with long follow-up periods would constitute a very expensive endeavour).

Another interpretation could be that individuals with specific phenotypes or highly severe forms of asthma do not respond to the treatment [47]. Consequently, their asthma symptoms may worsen despite the intake of preventive asthma mediaction. One could also argue that their disease status would even worsen faster if not having taken any medication. One should also take into account that patients with more severe symptoms are more likely to take preventive medicine in comparison to patients with less severe phenotypes. Another important aspect might be insufficient adherence to the treatment. Several studies have shown that asthma patients frequently do not use their medication as often as needed or not in the right manner [48–50]. This effect may especially occur when physicians do not apply recommended prescription guidelines properly or may have insufficient knowledge about preventive measures and diagnosis [51]. In this context, it also needs to be highlighted that our findings are in line with other studies observing low asthma control despite control medication use [15, 16, 52]. On the other hand, a recently published large-scale cohort study from the United States did observe a beneficial effect of asthma medication, specifically of inhaled corticosteroids [53]. What may also be taken into account is the fact that there is some evidence that the use of *ß*-agonists and other bronchodilators can lead to paradoxical bronchoconstriction [54]. To the best of our knowledge, however, such effects are mainly associated with short-acting *ß*-agonists which we classified as reliever medication [55]. Since our main exposure was the use of control medication, we thus expect any such effect to have a rather low impact on the results of our study.

We acknowledge that our study may have several limitations. Due to the small sample size of 121 asthmatic children at baseline, we could not divide the study population into subgroups for comparisons such as female/male or classification by asthma severity. We also had no classification of asthma severity beyond medication. We thus could not adjust our analyses for asthma severity so that we cannot rule out that our results are influenced by confounding due to disease severity. This could have biased our results in the way that participants with more severe asthma are also more likely to take control medication. Consequently, the risk increment that we found could be explained by reverse causation. Not taking into account sex differences could lead to ignoring potential effect modification of treatment effect by sex. Furthermore, also because of the sample size, we could not differentiate between different groups of exposure referring to different drugs. To be able to calculate the long-term effect of specific medications, future studies with larger sample sizes are thus needed. The binary classification of the exposure group could have also led to a violation of the above-mentioned consistency assumption, meaning that not all types of asthma medication have the same effect and not all asthmatic people respond similarly to the treatment. Nevertheless, when we performed sensitivity analyses for the exposure, the results seemed to be robust, demonstrating on the other hand the robustness of the TMLE approach. Moreover, by defining binary variables we could be more confident not violating the positivity assumption. Another limitation is that our outcome is not comparable to standardised measures of asthma control such as the Asthma Control Questionnaire (ACQ) [56] or the Asthma Control Test (ACT) [57] because it is based on data from the ISAAC study in which symptom frequency was asked for the last twelve months and not for 1 month. In this context, we also acknowledge that the time period between the baseline and the first follow-up was relativel large, and we cannot entirely be sure how many changes in their patterns of medication use some individuals may have had here and there. Comparison to other study populations should also be made with caution because our sample is restricted to children and young adults. Moreover, Vermeire and colleges found a variation in guideline adherence by doctors of different European countries [16]. Hence, generalisability to populations outside of Germany is limited. We assume that recall bias is not likely to have occurred because asthma is a chronic disease with regular treatment so that we assume participants to be able to correctly report if they currently had the disease or not. What concerns selection bias, former analyses of the SOLAR study have shown that this type of bias does not seem to play a major role in this study [58].

The strengths of our study are the prospective and observational design that provides a more realistic reflection of asthmatic children and adolescents than randomised controlled trials [13, 14]. Most notably, to our knowledge, this one of the first studies in this field to apply the statistical method of targeted maximum likelihood estimation. This tool is especially powerful to address the issue of time-varying confounding and treatment, thus being a suitable and up-to-date statistical approach and ensuring less bias by model miss specification and more power, despite a small sample size [19]. The results of the various analyses including the sensitivity analyses presented in the present manuscript confirm the usability and robustness of this method.

Altogether, our results suggest that treatment of asthma on a population level still can be optimised. Ideally, lung function should not decrease under treatment, but this was the case in our results. Additional targeted therapies may thus be needed for those patients who do not respond to treatment [59]. Moreover, intervention strategies, which elevate adherence to guidelines by patients and doctors might be measures to ensure that control medication has a more beneficial effect [60]. Finally, additional non-pharmacological interventions such as trigger-avoidance, smoking reduction or physical activity should be considered to improve symptoms of asthmatic individuals [6].

## Conclusion

While we could confirm the tagerted maximum likelihood estimation to be a usable and robust statistical tool, from a clinical perspectice we did not observe the desired beneficial effect of asthma control medication on asthma symptoms. Despite the suitability of our statistical methods, our results may, however, still be influenced by the potential impact of confounding by disease severity and additional limitations such as the small sample size or reverse causation. Our results should thus be interpreted with caution.

## Supplementary Information

**Additional file 1: **Characteristics of the study participants for the sensitivity analysis at the three time points ISAAC II, SOLAR I and SOLAR II (non-imputed data); *N* = 63.

**Additional file 2:.** Associations between asthma medication use and asthma symptoms at SOLAR I and SOLAR II obtained through TMLE.

**Additional file 3:.** Additive treatment effect of control medication on lung function, obtained through Marginal Structural Models.

**Additional file 4:.** Additive treatment effect of asthma medication on lung function, obtained through marginal structural models.

## Data Availability

The datasets used and/or analysed during the current study are available from the corresponding author on reasonable request.

## Abbreviations

ACQ: Asthma Control Questionnaire
ACT: Asthma Control Test
ATC: Anatomical Therapeutic Chemical classification system
BHR: Bronchial hyperresponsiveness
CI: Confidence interval
cRCTs: Clinical randomized controlled trials
ECRHS: European Community Respiratory Health Survey
FEV_1s_: Forced expiratory volume in one second
FVC: Forced vital capacity
GINA: Global Initiative for Asthma
ICS: Inhaled corticosteroids
ISAAC: International Study of Asthma and Allergies in Childhood
MSM: Marginal structural models
RR: Relative risk
SOLAR: Study on Occupational Allergy Risks
YLD: Years lived with disability
