# Supplementary material for: Long-term effects of asthma medication on asthma symptoms: an application of the targeted maximum likelihood estimation
Source: BMC Med Res Methodol. 2020 Dec 16;20:307. doi: 10.1186/s12874-020-01175-9 (PMC7739451; doi:10.1186/s12874-020-01175-9)
Supplement: Supplementary file 1 — Additional file 1: Characteristics of the study participants for the sensitivity analysis at the three time points ISAAC II, SOLAR I and SOLAR II (non-imputed data); N = 63. [file 12874_2020_1175_MOESM1_ESM.docx]

**Additional file 1.** Characteristics of the study participants for the sensitivity analysis at the three time points ISAAC II, SOLAR I and SOLAR II (non-imputed data); N=63.

| **Variables** | **ISAAC II** | **SOLAR I** | **SOLAR II** |
| --- | --- | --- | --- |
| Age, mean (sd^1^) | 9.6 (0.56) | 17.0 (0.62) | 22.3 (0.71) |
| NAs^2^ | 0 | 0 | 0 |
| Height [cm], mean (sd) | 143.4 (6.6) | 175.4 (9.1) | 176.7 (10.0) |
| NAs, N (%) | 0 | 0 | 0 |
| FEV_1s_ / FVC [%], mean (sd) | 85.4 (6.6) | n.m.^3^ | 81.7 (6.8) |
| NAs, N (%) | 0 |  | 24 (38.1) |
| Asthma symptoms, N (%) |  |  |  |
| yes | 62 (98.4) | 39 (61.9) | 39 (61.9) |
| NAs | 0 | 0 | 1 (1.6) |
| Control medication, N (%) |  |  |  |
| yes | 34 (54.0) | 9 (14.3) | n.m. |
| NAs | 9 (14.3) | 1 (1.6) |  |
| Only reliever medication, N (%) |  |  |  |
| Yes | 10 (15.9) | 17 (27.0) | n.m. |
| NAs | 9 (14.3) | 1 (1.6) |  |
| No medication intake, N (%) |  |  |  |
| Yes | 10 (15.9) | 36 (57.1) | n.m. |
| NAs | 9 (14.3) | 1 (1.6) |  |
| Study center, N (%) |  |  |  |
| Munich | 37 (58.7) |  |  |
| NAs | 0 |  |  |
| Sex, N (%) |  |  |  |
| male | 41 (65.1) |  |  |
| NAs | 0 |  |  |
| SES^4^ parents, N (%) |  |  |  |
| high | 34 (54.0) |  |  |
| NAs | 1 (1.6) |  |  |
| Asthma history of parents^5^, N (%) |  |  |  |
| yes | 13 (20.6) |  |  |
| NAs | 9 (14.3) |  |  |
| Passive smoking^6^, N (%) |  |  |  |
| yes | 18 (28.6) | 36 (57.1) | 29 (46.0) |
| NAs | 0 | 0 | 0 |
| Current smoker^7^, N (%) |  |  |  |
| yes | n.m. | 21 (33.3) | 26 (41.3) |
| NAs |  | 0 | 0 |
| Physical activity^8^, N (%) |  |  |  |
| yes | n.m. | 48 (76.2) | 41 (76.2) |
| NAs |  | 0 | 0 |
| Obesity^9^, N (%) |  |  |  |
| yes | n.m. | 5 (7.9) | 16 (25.4) |
| NAs |  | 1 (1.6) | 0 |
| Current hayfever^10^, N (%) |  |  |  |
| yes | 12 (19.0) | 17 (27.0) | 17 (27.0) |
| NAs | 0 | 1 (1.6) | 1 (1.6) |

**Abbreviations and comments. ^1^**Sd = standard deviation; ^2^NAs = missing values; ^3^n.m. = not measured; ^4^SES = socio-economic status (high if one parent has at least higher secondary education or university degree); ^5^Yes if at least one parent has ever had asthma; ^6^ISAAC II: passive smoking of child if parents are current smokers, SOLAR I/SOLAR II: Yes if exposition to passive smoke per day > 0,5 hours; ^7^Current smoker = smoked ever for 1 year and smoked in the last month; ^8^Yes if participant reports doing sport at least once per week; ^9^Yes if body mass index >=25; ^10^Yes if ever diagnosed hay fever by a doctor and itchy eyes and runny nose without cold in the last 12 months.
