# Supplementary material for: Long-term effects of asthma medication on asthma symptoms: an application of the targeted maximum likelihood estimation
Source: BMC Med Res Methodol. 2020 Dec 16;20:307. doi: 10.1186/s12874-020-01175-9 (PMC7739451; doi:10.1186/s12874-020-01175-9)
Supplement: Supplementary file 2 — Additional file 2:. Associations between asthma medication use and asthma symptoms at SOLAR I and SOLAR II obtained through TMLE. [file 12874_2020_1175_MOESM2_ESM.docx]

**Additional file 2.** Associations between asthma medication use and asthma symptoms at SOLAR I and SOLAR II obtained through TMLE.

| **Treatment scenario (asthma medication)** | **Adjusted relative risk to report asthma symptoms at SOLAR I (95% CI)** | **Adjusted relative risk to report asthma symptoms at SOLAR II (95% CI)** |
| --- | --- | --- |
| Intervention 3 vs. Intervention 1  (1,1) (0,0) | 1.33 (0.97; 1.69) | 1.27 (0.87; 1.66) |
| Intervention 3 vs. No Intervention  (1,1) | 1.29 (0.92; 1.66) | 1.19 (0.73; 1.64) |
| Intervention 3 vs. Intervention 2  (1,1) (1,0) | 1.61 (1.32; 1.90) | 1.32 (1.03; 1.62) |
| Intervention 2 vs. Intervention 1  (1,0) (0,0) | 0.84 (0.41; 1.27) | 0.95 (0.51; 1.39) |
| Intervention 2 vs. No Intervention  (1,0) | 0.79 (0.45; 1.14) | 0.89 (0.44; 1.34) |
