# Supplementary material for: Long-term effects of asthma medication on asthma symptoms: an application of the targeted maximum likelihood estimation
Source: BMC Med Res Methodol. 2020 Dec 16;20:307. doi: 10.1186/s12874-020-01175-9 (PMC7739451; doi:10.1186/s12874-020-01175-9)
Supplement: Supplementary file 3 — Additional file 3:. Additive treatment effect of control medication on lung function, obtained through Marginal Structural Models. [file 12874_2020_1175_MOESM3_ESM.docx]

**Additional file 3.** Additive treatment effect of control medication on lung function, obtained through Marginal Structural Models.

|  | **Outcome lung function (FEV_1s_ / FVC in %)** |
| --- | --- |
| **Treatment scenario (control medication)** | **Adjusted additive treatment effect (95%CI) (mean difference between treatment scenarios)** |
| Intervention 3 vs. Intervention 1  (1,1) (0,0) | -3.02 (-6.36; 0.31) |
| Intervention 3 vs. No Intervention  (1,1) | -3.01 (-6.19; 0.16) |
| Intervention 3 vs. Intervention 2  (1,1) (1,0) | -4.48 (-6.97; -1.99) |
| Intervention 2 vs. Intervention 1  (1,0) (0,0) | 1.58 (-1.67; 4.84) |
| Intervention 2 vs. No Intervention  (1,0) | 1.73 (-1.84; 5.31) |
