# Supplementary material for: Long-term effects of asthma medication on asthma symptoms: an application of the targeted maximum likelihood estimation
Source: BMC Med Res Methodol. 2020 Dec 16;20:307. doi: 10.1186/s12874-020-01175-9 (PMC7739451; doi:10.1186/s12874-020-01175-9)
Supplement: Supplementary file 4 — Additional file 4:. Additive treatment effect of asthma medication on lung function, obtained through marginal structural models. [file 12874_2020_1175_MOESM4_ESM.docx]

**Additional file 4.** Additive treatment effect of asthma medication on lung function, obtained through marginal structural models.

|  | **Outcome lung function (FEV1/FVC in %)** |
| --- | --- |
| **Treatment scenario (asthma medication)** | **Adjusted additive treatment effect (95 %CI) (mean difference between treatment scenarios)** |
| Intervention 3 vs. Intervention 1  (1,1) (0,0) | -3.84 (-8.17; 0.49) |
| Intervention 3 vs. No Intervention  (1,1) | -1.67 (-6.53; 3.19) |
| Intervention 3 vs. Intervention 2  (1,1) (1,0) | -2.82 (-4.93; -0.71) |
| Intervention 2 vs. Intervention 1  (1,0) (0,0) | -0.97 (-5.13; 3.19) |
| Intervention 2 vs. No Intervention  (1,0) | 1.07 (-4.89; 7.03) |
